# Kidney Transplant Function in Recipients from Deceased Donors with COVID-19

**DOI:** 10.3390/jcm15134955

**Published:** 2026-06-25

**Authors:** Mengmeng Ji, Dema Yaseen Alsabbagh, Siobhan Sutcliffe, Massini Merzkani, Krista L. Lentine, Bekir Tanriover, Su-Hsin Chang, Tarek Alhamad

**Affiliations:** 1 Division of Public Health Sciences, Department of Surgery, Washington University School of Medicine in St. Louis, St. Louis, MO 63110, USA; j.mengmeng@wustl.edu (M.J.); sutcliffes@wustl.edu (S.S.); chang.su-hsin@wustl.edu (S.-H.C.); 2 Division of Nephrology, Washington University School of Medicine in St. Louis, 660 Euclid Ave., Mail Stop 8126-05-06, St. Louis, MO 63110, USA; dema@wustl.edu (D.Y.A.); massini@wustl.edu (M.M.); 3 Division of Nephrology, Saint Louis University, St. Louis, MO 63104, USA; krista.lentine@health.slu.edu; 4 Division of Nephrology, University of Arizona, Tucson, AZ 85719, USA; btanriover@arizona.edu

**Keywords:** kidney transplant, estimated glomerular filtration rate, eGFR, COVID-19, deceased donor

## Abstract

**Background:** During the COVID-19 pandemic, uncertainty regarding the safety of kidneys from COVID-19-positive donors led to a reduction in kidney transplants and increased organ non-use in the United States. This study aims to evaluate whether donor COVID-19 positivity is associated with one-year post-transplant estimated glomerular filtration rate (eGFR) among kidney transplant recipients. **Methods:** This retrospective cohort study used data from the United States Organ Procurement and Transplantation Network (OPTN) 2020–2024. Donor COVID-19 status was determined by the SARS-CoV-2 nucleic acid amplification technique (NAT) and antibody test results. The main outcome was recipients’ one-year eGFR, estimated by the CKD-EPI 2021 formula. Linear regression models were used to compare the mean one-year eGFR among donor COVID-19 groups, adjusted by inverse probability of treatment weights. Interaction terms of donor acute kidney injury status and race were assessed to evaluate effect modification. **Results:** Among 38,199 included kidney transplant recipients, 1090 (2.9%) received kidneys from donors with active COVID-19 infection, 423 (1.1%) from donors with resolved infection, and 36,686 (96.0%) from COVID-19-negative donors. After weighting and adjustment, there was no significant difference in one-year eGFR for recipients of kidneys from donors with active COVID-19 (mean difference, 0.05 [95% CI, −1.08 to 1.18]) or resolved infection (mean difference, −0.27 [95% CI, −2.19 to 1.64]) compared with COVID-19-negative donors. Neither donor AKI nor donor race modified the association between donor COVID-19 status and one-year eGFR. **Conclusions:** This study suggests that kidneys from COVID-19-positive donors may be a viable option without compromising short-term allograft function as measured by 1-year eGFR.

## 1. Introduction

The COVID-19 pandemic from 2020 to 2023 led to a substantial decrease in the number of kidney transplants performed and an increase in the number of kidneys discarded in the United States [1,2]. This was driven by multiple factors, including concerns about the potential transmission of COVID-19 to uninfected recipients and uncertainties about the kidney transplant function [3,4,5,6,7,8,9,10,11].

While there is more utislization of kidneys from donors with positive COVID-19 in the later phases of the pandemic, there are ongoing concerns about the function of kidney allografts. Two small cohort studies have noted worse renal allograft function (measured in estimated glomerular filtration rate, eGFR) in recipients of kidneys from COVID-19-positive donors [12,13]. However, reduced early eGFR was observed only when the donor’s cause of death was COVID-19 whereas kidneys from COVID-19-positive donors who died of other causes showed comparable function to those from COVID-19-negative donors [12,13]. Several small single-center case series and reports have described eGFR outcomes for kidneys from COVID-19-positive deceased donors [14,15,16,17,18]. Although early delayed graft function was occasionally observed, mid-term outcomes, including serum creatinine and eGFR at 6–9 months, were generally favorable without evidence of donor-derived viral transmission. However, most of these studies were limited by very small sample sizes (often <15 recipients) and short follow-up.

In patients with COVID-19, there have been reports of collapsing glomerulopathy in native kidneys [19,20,21,22,23]. But it remains unclear how many COVID-19-positive deceased donors may have experienced this process and whether a “transient” acute kidney injury would impact graft outcomes post-transplant. Additionally, donor race has been shown to influence post-transplant outcomes [24], which may modify the relationship between COVID-19 positivity and graft function.

In the current study, we evaluated the impact of transplantation from COVID-19-positive deceased donors on recipient eGFR one-year post-transplant in a large cohort of kidney transplant recipients. We also examined whether AKI in donors or donor race moderates the relationship between COVID-19 positivity and recipient eGFR. Using data from a national transplant registry, we aim to provide a robust and comprehensive exam of graft function in recipients of kidneys from COVID-19-positive donors at one-year post-transplant.

## 2. Methods

### 2.1. Study Population and Design

The data was obtained from the June 2024 version of the OPTN Standard Analysis and Research File. We conducted a retrospective cohort analysis of all adult patients aged ≥ 18 years who received their first kidney transplant from a deceased donor between 3/2020, the onset of the COVID-19 epidemic in the United States, and 6/2023. The end date was chosen to ensure at least one year of post-transplant follow-up. Exclusion criteria for recipients included (1) younger than 18 years at the time of transplant, (2) multiorgan transplant (e.g., pancreas-kidney transplant), (3) previous KT, (4) missing data on serum creatinine level around one-year post-transplant for calculation of eGFR, and (5) missing data on donor COVID-19 status at the time of transplant.

This study used publicly available, de-identified data from the OPTN and thus was deemed exempt from institutional review board (IRB) review by Washington University in St. Louis. The requirement for written informed consent was waived.

### 2.2. Donor COVID-19 Status

SARS-CoV-2 nucleic acid amplification technique (NAT) and antibody test results, as well as the time (in days) between the test specimen collection and kidney recovery dates, were used to determine donor COVID-19 status, per previous methods [7]. A positive SARS-CoV-2 NAT result within 7 days before procurement was defined as an active COVID-19 infection (COVID-19-positive). Donors who had a positive SARS-CoV-2 NAT result one week or longer before procurement or a positive antibody test result were considered to have a resolved COVID-19 infection.

### 2.3. One-Year Post-Transplant eGFR

One-year post-transplant eGFR was estimated by the CKD-EPI 2021 formula, a race-free equation based on patient age, gender, and serum creatinine [25]. Because the timing of patient visits may vary, we allowed for a window of ±2 months from patients’ one-year anniversary to estimate their 1-year post-transplant eGFR.

### 2.4. Covariates

Donor and recipient covariates were obtained from the OPTN database. Donor factors included: age, gender (female, male), race/ethnicity (Hispanic, non-Hispanic Black, Non-Hispanic White, Others), body mass index (BMI; <18.5, 18.5–24.9, 25.0–29.9, ≥30.0 kg/m^2^), hypertension status (yes, no), donation after cardiac death (yes, no), kidney donor profile index (KDPI; ≤20, 21–35, 36–84, and ≥85%), cause of death (anoxia, cerebrovascular/stroke, head trauma, and central nervous system tumor), risk factors for blood-borne disease transmission (yes, no), and AKI (defined as creatinine ≥ 2 mg/dL) at the time of transplant [26].

Recipient factors included: age, gender, race/ethnicity, BMI, history of hypertension, diabetes mellitus status, panel reactive antibodies (PRA; 0, 0–19, 20–80, >80%), duration of dialysis (0, 1 to 24, 25–60, >60 months), and cytomegalovirus status (CMV).

### 2.5. Statistical Analyses

Summary statistics on donor and recipient characteristics stratified by donor COVID-19 status were calculated. The association between donor COVID-19 status (active, resolved, and negative) and one-year eGFR was estimated by linear regression. Potential confounding was addressed using inverse probability of treatment weights (IPTWs) and adjusting for all donor and recipient factors listed in the covariate section. IPTWs were derived by multinomial logistic regression, including all donor factors listed above. Weights were stabilized by multiplying by the prevalence of each donor COVID-19 category and truncated at the 5th percentile and 95th percentiles to minimize the influence of extreme weights and improve the stability of weighted estimates [20]. We also performed stratified analyses by donor race (non-Hispanic Black versus non-Hispanic White) and donor AKI.

Missing data were handled based on the proportion of missingness, with observations excluded for variables containing <5% missing values and a separate “missing” category incorporated into propensity score and outcome models for variables with ≥5% missingness. All tests were two-sided. *p* values < 0.05 were considered statistically significant. All analyses were performed using Python (version 3.12.0; Python Software Foundation, Wilmington, DE, USA).

## 3. Results

**Figure 1:**
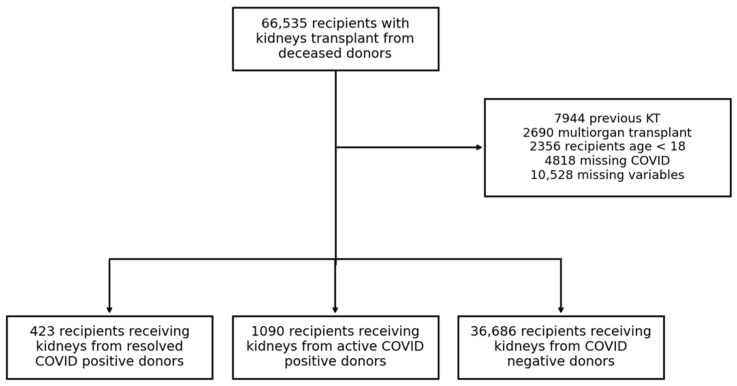
Flowchart of study cohort.

In total, 66,535 patients received a kidney transplant between 1 March 2020 and 30 June 2023. Among them, 18,404 received kidneys from living donors, 7944 had a previous kidney transplant, 2690 received multiple organs at the time of transplant, 2356 were younger than 18 years old, 4818 had missing data on donor COVID-19 status, and 10,528 had missing one-year follow-up data. Therefore, 38,199 KT recipients from deceased donors were included in the analysis, among which 1090 (2.9%) were from donors with active COVID-19 infection, 423 (1.1%) were from donors with resolved COVID-19 infection, and 36,686 (96.0%) were from COVID-19-negative donors (Figure 1).

Table 1 summarizes the characteristics of kidney donors and recipients by donor COVID-19 infection status. Compared to COVID-19-negative donors, positive donors were more likely to be male, to have obesity, and to have died a cardiac death. They were also less likely to have hypertension, a high creatinine value, a high KDPI, and donor risk factors for blood transfusion. In addition, donors with active COVID-19 infection tended to be younger, and those with resolved infection tended to be older than negative donors. Donors with resolved infection were also more likely to be Hispanic and to have diabetes than negative donors. Compared to recipients who received kidneys from COVID-19-negative donors, recipients of kidneys from COVID-19-positive donors were more likely to be non-Hispanic White, male, and not have received dialysis. The baseline characteristics of recipients included in the analysis and those excluded because of missing 1-year eGFR are presented in Table S1.

**Figure 2:**
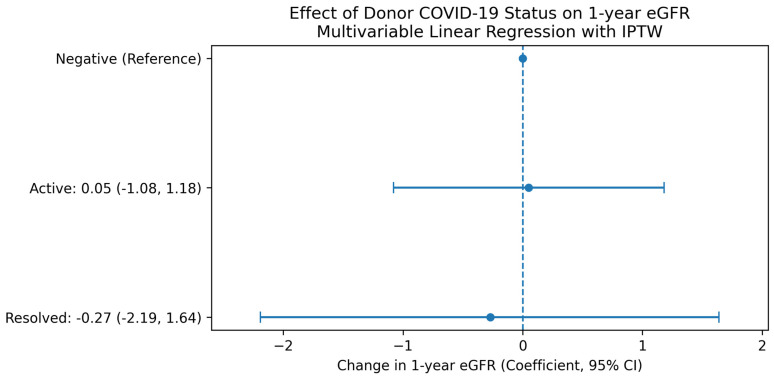
Association between donor COVID-19 status and 1-year estimated glomerular filtration rate (eGFR) in IPTW-adjusted multivariable linear regression models. The blue circles represent the estimated regression coefficients (mean differences in 1-year eGFR compared with recipients of kidneys from COVID-19-negative donors). The horizontal blue lines indicate the corresponding 95% confidence intervals. The multivariable model adjusted for donor characteristics, including COVID-19 status, age, race/ethnicity, sex, body mass index (BMI), hypertension, diabetes, serum creatinine, kidney donor profile index (KDPI), cause of death, and HIV risk, as well as recipient characteristics, including age, race/ethnicity, sex, BMI, diabetes, cytomegalovirus (CMV) serostatus, dialysis duration, and panel reactive antibody (PRA) level.

In unadjusted analyses, recipients of kidneys from donors with COVID-19 infection had a significantly lower mean one-year eGFR (mean eGFR: 61.0 for active infection and 57.9 for resolved infection) than those receiving kidneys from COVID-19-negative donors (mean eGFR: 59.7; *p* < 0.001). In the IPTW-adjusted analysis, recipients of kidneys from donors with active COVID-19 infection showed no significant difference in one-year eGFR (mean difference, 0.05; 95% CI, −1.08 to 1.18; *p* = 0.933). Similarly, for recipients of kidneys from donors with resolved COVID-19 infection, the reduction in one-year eGFR was not statistically significant (mean difference, −0.27; 95% CI, −2.19 to 1.64; *p* = 0.779; Figure 2). Compared with kidneys from non-Hispanic White donors, kidneys from non-Hispanic Black donors were associated with a modestly lower one-year eGFR (mean difference, −0.68 mL/min/1.73 m^2^, 95% CI: −1.30 to −0.07; *p* = 0.03). No significant differences were observed for kidneys from Hispanic donors (mean difference, −0.19, 95% CI: −0.76 to 0.38; *p* = 0.51) or donors of other racial/ethnic groups (mean difference, −0.70, 95% CI: −1.67 to 0.27; *p* = 0.16). Covariate balance before and after IPTW is presented in Table S2. The complete IPTW-adjusted multivariable linear regression model is presented in Table S3.

In additional analyses, we fitted a linear model including interaction terms (AKI × donor COVID-19 status and donor race × donor COVID-19 status). Neither interaction was statistically significant, indicating no evidence that the effect of AKI or donor race on 1-year eGFR varies by donor COVID-19 status.

## 4. Discussion

In this retrospective cohort study, we found that recipients of kidneys from deceased donors with active or resolved COVID-19 infection had similar one-year eGFR compared with recipients of kidneys from COVID-19-negative donors. We found no adverse impact on one-year eGFR outcomes among recipients of kidneys from COVID-19-positive donors with AKI compared with those without AKI. Our analysis found that recipients of kidneys from Black donors had significantly lower one-year eGFR compared to recipients of kidneys from White donors. However, donor race did not moderate the impact of donor COVID-19 positivity on one-year eGFR.

A previous study from our group found that kidneys from donors with active or resolved COVID-19 infection were more likely to be discarded before 2023, but since then have no longer been associated with increased risk of non-use, reflecting an evolving understanding of their safety and viability [7]. However, even in the post-pandemic era, continued assessment of COVID-19’s impact is warranted. WHO’s dashboard shows low overall activity but regional upticks and ongoing hospitalizations/deaths, primarily among older adults. CDC’s R_t_ estimates indicate growth in many U.S. states, and WHO’s updates highlight continued variant changes [27,28]. Findings from this study provide practical reassurance to transplant programs that accepting kidneys from donors with active or resolved COVID-19 does not compromise short-term graft function and supports organ-utilization policies aimed at maximizing utilization of available kidneys, which remains critical in the context of organ shortages [29].

It was hypothesized that recipients of kidneys from COVID-19-positive donors would experience a reduction in eGFR, potentially due to factors such as inflammatory responses, hypoxia, or renal microvascular injury, all of which can impair kidney function [30,31]. However, our study findings suggest that the impact of donor COVID-19 infection on short-term kidney function is minimal. This is consistent with other studies showing minimal long-term effects on graft function from COVID-19-positive donors, though continued monitoring is essential as the long-term outcomes of such transplants remain uncertain [7].

Recent studies have raised concerns about the impact of COVID-19 infection in deceased kidney donors, particularly with respect to AKI and the APOL1 high-risk genotype. Wu et al. found that COVID-19-positive donors with an *APOL1* high-risk genotype had a higher risk of developing AKI, suggesting a potential “double-hit” effect on kidney function, whereby both COVID-19 infection and the *APOL1* genotype exacerbate kidney injury [22,23,32,33,34,35]. Studies such as the one conducted by Hall et al. have not observed significant differences in 6-month eGFR outcomes by donor AKI status, suggesting that kidneys from donors with AKI can still have acceptable short-term graft function [34]. Many studies have also demonstrated that kidneys from deceased donors with pre-transplant AKI can have good long-term graft survival [21,22,34]. Our study adds to the literature by investigating the impact of AKI on the association between COVID-19 positivity and one-year eGFR outcomes, demonstrating no significant moderation of this association.

Donor race has also been found to influence kidney transplant outcomes, with some studies suggesting that kidneys from Black donors may be associated with a higher risk of graft loss compared to those from White donors [24,36,37,38]. In this analysis, kidneys from non-Hispanic Black donors were associated with a modestly lower one-year eGFR compared with kidneys from non-Hispanic White donors. However, these racial disparities may be driven more by socio-economic factors, access to healthcare, and other social determinants of health, rather than inherent biological differences. Our analysis found that recipients of kidneys from Black donors had significantly lower one-year eGFR compared to recipients of kidneys from White donors. However, donor race did not moderate the impact of donor COVID-19 positivity on one-year eGFR.

This study has several notable strengths. First, we utilized a comprehensive national transplant registry that captures detailed and reliable information on all organ donations and transplants across the United States, ensuring a robust and representative dataset. Our analysis included kidney transplants performed between March 2020 and June 2023. This timeline provides valuable insights into the experiences of recipients during the early years of the pandemic, when risks were likely higher due to limited understanding of COVID-19 and lower population immunity. Second, our study addresses potential selection bias by employing a rigorous matching approach and standardizing differences between donor groups, enhancing the validity of our comparisons. Third, unlike previous studies with shorter follow-up periods, our analysis includes one-year post-transplant data, allowing for a more thorough evaluation of graft function over time. Additionally, we explored key factors such as AKI and donor race as potential moderators, providing novel insights into the impact of COVID-19 on graft outcomes.

The study has several limitations. Due to the lack of a clear timeline for SARS-CoV-2 infection among donors, we determined active or resolved status based on the test collection and donor recovery dates. Donor AKI was defined using terminal serum creatinine ≥ 2 mg/dL because the OPTN STAR database lacks the serial creatinine and urine output measurements required for KDIGO classification. Thus, some degree of AKI misclassification is possible, which may have resulted in residual confounding. This study was observational and thus can identify associations but not establish causality. Selection bias may have occurred based on whether candidates chose to receive kidneys from COVID-19-positive donors, as well as physician donor and candidate selection practices. While we attempted to address potential bias by using propensity score weightings to improve covariate balance across the groups, residual confounding may still exist based on decision making surrounding receipt of kidneys from COVID-19-positive donors. The OPTN database does not capture several potentially relevant donor COVID-19 characteristics, including symptom severity, viral load or cycle threshold values, duration of hospitalization before procurement, or whether COVID-19 directly contributed to donor death. These unmeasured factors may influence organ quality and transplant outcomes and could contribute to residual confounding. Furthermore, the dataset does not contain detailed match-run or offer-level information; therefore, potential confounding related to allocation practices and organ acceptance patterns could not be fully assessed.

## 5. Conclusions

This cohort analysis demonstrates comparable one-year graft function among recipients of kidneys from deceased donors with active, resolved, and negative COVID-19. These data support that transplanting kidneys from COVID-19-positive deceased donors appears to be safe in the short-term with similar eGFR. However, further research is needed to evaluate long-term outcomes as more data become available. These data are relevant as COVID-19 transitions to an endemic infection with ongoing waves driven by new variants, waning immunity, and vaccination patterns.

## Figures and Tables

**Table 1:** Characteristics of kidney donors and recipients by donor COVID-19 infection status.

Clinical Factors	Active COVID-19 Infection	Resolved COVID-19 Infection	Non-COVID-19 Infected	Total
N	1090	423	36,686	38,199
Donor characteristics				
Age (Mean [SD]) ^a,b^	37.4 (14.1)	42.0 (13.6)	39.7 (14.6)	39.7 (14.6)
Race ^a^				
Hispanic	759 (69.6%)	260 (61.5%)	24,736 (67.4%)	25,755 (67.4%)
Non-Hispanic Black	122 (11.2%)	62 (14.7%)	5052 (13.8%)	5236 (13.7%)
Non-Hispanic White	171 (15.7%)	84 (19.9%)	5422 (14.8%)	5677 (14.9%)
Other	38 (3.5%)	17 (4.0%)	1476 (4.0%)	1531 (4.0%)
Gender				
Female	363 (33.3%)	153 (36.2%)	13,196 (36.0%)	13,712 (35.9%)
Male	727 (66.7%)	270 (63.8%)	23,490 (64.0%)	24,487 (64.1%)
BMI, kg/m^2 a,b^				
<18.5	48 (4.4%)	16 (3.8%)	1462 (4.0%)	1526 (4.0%)
18.5–24.9	335 (30.7%)	87 (20.6%)	10,982 (29.9%)	11,404 (29.9%)
25–29.9	290 (26.6%)	101 (23.9%)	10,979 (29.9%)	11,370 (29.8%)
≥30	417 (38.3%)	219 (51.8%)	13,263 (36.2%)	13,899 (36.4%)
Diabetes status ^a^				
No	1018 (93.4%)	374 (88.4%)	33,656 (91.7%)	35,048 (91.8%)
Yes	72 (6.6%)	49 (11.6%)	3030 (8.3%)	3151 (8.3%)
Hypertension status ^b^				
No	838 (76.9%)	310 (73.3%)	26,208 (71.4%)	27,356 (71.6%)
Yes	252 (23.1%)	113 (26.7%)	10,478 (28.6%)	10,843 (28.4%)
Creatinine (mg/dL) ^a,b^				
<1.0	612 (56.2%)	319 (75.4%)	19,655 (53.6%)	20,586 (53.9%)
1.0–1.5	250 (22.9%)	56 (13.2%)	8426 (23.0%)	8732 (22.9%)
>1.5	228 (20.9%)	48 (11.4%)	8605 (23.5%)	8881 (23.3%)
Donation after cardiac death ^a,b^				
No	62.7%	35.0%	70.6%	70.2%
Yes	37.3%	65.0%	29.4%	29.8%
Cause of death ^a,b^				
Anoxia	538 (49.4%)	196 (46.3%)	18,383 (50.1%)	19,117 (50.1%)
Cerebrovascular	179 (16.4%)	62 (14.7%)	7537 (20.5%)	7778 (20.4%)
Head Trauma	262 (24.0%)	69 (16.3%)	9642 (26.3%)	9973 (26.1%)
Other	111 (10.2%)	96 (22.7%)	1124 (3.1%)	1331 (3.5%)
Kidney Donor Profile Index (KDPI) ^b^				
≤0.2	350 (32.1%)	105 (24.8%)	9452 (25.8%)	9907 (25.9%)
>0.2 to ≤0.35	250 (22.9%)	88 (20.8%)	7412 (20.2%)	7750 (20.3%)
>0.35 to ≤0.85	461 (42.3%)	215 (50.8%)	17,926 (48.9%)	18,602 (48.7%)
>0.85	29 (2.7%)	15 (3.6%)	1896 (5.2%)	1940 (5.1%)
Risk factors for blood transfusion ^b^				
No	864 (79.3%)	346 (81.8%)	28,107 (76.6%)	29,317 (76.8%)
Yes	226 (20.7%)	77 (18.2%)	8579 (23.4%)	8882 (23.3%)
Recipient characteristics				
Age (Mean [SD]) ^a^	53.3 (13.5)	55.4 (12.7)	54.0 (13.2)	54.0 (13.3)
Race ^b^				
Hispanic	418 (38.4%)	169 (40.0%)	13,047 (35.6%)	13,634 (35.7%)
Non-Hispanic Black	363 (33.3%)	121 (28.6%)	12,187 (33.2%)	12,671 (33.2%)
Non-Hispanic White	218 (20.0%)	89 (21.0%)	7579 (20.7%)	7886 (20.6%)
Others	91 (8.4%)	44 (10.4%)	3873 (10.6%)	4008 (10.5%)
Gender				
Female	416 (38.2%)	167 (39.5%)	14,646 (39.9%)	15,229 (39.9%)
Male	674 (61.8%)	256 (60.5%)	22,040 (60.1%)	22,970 (60.1%)
BMI, kg/m^2^				
<18.5	15 (1.4%)	7 (1.7%)	542 (1.5%)	564 (1.5%)
18.5–24.9	270 (24.8%)	122 (28.8%)	9700 (26.4%)	10,092 (26.4%)
25–29.9	375 (34.4%)	152 (35.9%)	12,171 (33.2%)	12,698 (33.2%)
≥30	430 (39.5%)	142 (33.6%)	14,273 (38.9%)	14,845 (38.9%)
Diabetes status ^a^				
No	672 (61.7%)	241 (57.0%)	22,176 (60.5%)	23,089 (60.4%)
Yes	418 (38.4%)	182 (43.0%)	14,510 (39.6%)	15,110 (39.6%)
Duration of dialysis ^b^				
No	174 (16.0%)	90 (21.3%)	5384 (14.7%)	5648 (14.8%)
≤24 months	257 (23.6%)	93 (22.0%)	7830 (21.3%)	8180 (21.4%)
25–60	403 (37.0%)	130 (30.7%)	11,904 (32.5%)	12,437 (32.6%)
>60	256 (23.5%)	110 (26.0%)	11,568 (31.5%)	11,934 (31.2%)
PRA				
0	636 (58.4%)	243 (57.5%)	21,029 (57.3%)	21,908 (57.4%)
>0 to ≤20	157 (14.4%)	60 (14.2%)	4737 (12.9%)	4954 (13.0%)
>20 to ≤80	188 (17.3%)	74 (17.5%)	6605 (18.0%)	6867 (18.0%)
>80	109 (10.0%)	46 (10.9%)	4315 (11.8%)	4470 (11.7%)
Post-kidney transplant 1-year eGFR (Mean [SD]) ^a^	61.0 (22.0)	57.9 (23.1)	59.7 (21.8)	59.7 (21.9)
CMV status				
Low (D−/R−)	147 (13.5%)	63 (14.8%)	4674 (12.7%)	4884 (12.8%)
Moderate (R+)	717 (65.8%)	278 (65.7%)	24,030 (65.5%)	25,025 (65.5%)
High (D+/R−)	209 (19.2%)	78 (18.4%)	7323 (20.0%)	7609 (19.9%)
Missing	18 (1.7%)	5 (1.2%)	649 (1.8%)	672 (1.8%)

^a^. Statistically significant difference between active COVID-19 infection and resolved COVID-19 infection groups (*p* < 0.05). ^b^. Statistically significant difference between any COVID-19 infection (active or resolved) and non-COVID-19 infected groups (*p* < 0.05). Abbreviations: BMI, body mass index; CMV, cytomegalovirus; and PRA, panel reactive antibody.

## Data Availability

The data presented in this study are openly available in OPTN at https://www.hrsa.gov/optn?from=optn.transplant.hrsa.gov (accessed on 2 August 2024).

## Supplementary Materials

The following supporting information can be downloaded at: https://www.mdpi.com/article/10.3390/jcm15134955/s1, Table S1. Characteristics of kidney donors and recipients for excluded because of missing 1-year eGFR; Table S2. Covariate Balance After IPTW; Table S3. Full IPTW-adjusted multivariable linear regression results for 1-year eGFR.
