# Supplementary material for: Kidney Transplant Function in Recipients from Deceased Donors with COVID-19
Source: J Clin Med. 2026 Jun 25;15(13):4955. doi: 10.3390/jcm15134955 (PMC13361632; doi:10.3390/jcm15134955)
Supplement: Supplementary file 1 [file jcm-15-04955-s001.zip › jcm-4369097-supplementary.pdf]

Supplementary Table S1. Characteristics of kidney donors and recipients for excluded because of missing 1-year eGFR

Clinical factors	Excluded	Included	p-value
N	4952	38199	
Donor characteristics			
Age (Mean, SD)	40.0 (14.2)	39.7 (14.6)	0.163
Race			0.007
Hispanic	780 (15.8%)	5677 (14.9%)	
Non-Hispanic Black	749 (15.1%)	5236 (13.7%)	
Non-Hispanic White	3224 (65.1%)	25755 (67.4%)	
Other	199 (4.0%)	1531 (4.0%)	
Gender			0.183
Female	1826 (36.9%)	13712 (35.9%)	
Male	3126 (63.1%)	24487 (64.1%)	
BMI, kg/m2			0.832
<18.5	202 (4.1%)	1526 (4.0%)	
18.5 - 24.9	1448 (29.2%)	11404 (29.9%)	
25 - 29.9	1493 (30.2%)	11370 (29.8%)	
≥30	1809 (36.5%)	13899 (36.4%)	
Diabetes status			0.664
No	4534 (91.6%)	35048 (91.8%)	
Yes	418 (8.4%)	3151 (8.3%)	
Hypertension status			0.289
No	3510 (70.9%)	27356 (71.6%)	
Yes	1442 (29.1%)	10843 (28.4%)	
Creatinine (mg/dl)			0.640
<1.0	2695 (54.4%)	20586 (53.9%)	
1.0 - 1.5	1103 (22.3%)	8732 (22.9%)	
>1.5	1154 (23.3%)	8881 (23.3%)	
Donation after cardiac death			0.224
No	3423 (69.1%)	26731 (70.0%)	
Yes	1529 (30.9%)	11468 (30.0%)	
Cause of death			0.093
Anoxia	2429 (49.1%)	19117 (50.1%)	
Cerebrovascular	1081 (21.8%)	7778 (20.4%)	
Head Trauma	1262 (25.5%)	9973 (26.1%)	
Other	180 (3.6%)	1331 (3.5%)	
Kidney Donor Profile Index (KDPI)			0.040
≤0.2	1226 (24.8%)	9907 (25.9%)	
>0.2 to ≤0.35	956 (19.3%)	7750 (20.3%)	
>0.35 to ≤0.85	2504 (50.6%)	18602 (48.7%)	
>0.85	266 (5.4%)	1940 (5.1%)	
Risk factors for blood transfusion			<0.001
No	3861 (78.0%)	29317 (76.8%)	
Yes	1026 (20.7%)	8882 (23.3%)	
Recipient characteristics			
Age (Mean, SD)	53.7 (12.9)	54.0 (13.3)	0.125
Race			<0.001

Hispanic	1008 (20.6%)	13634 (35.7%)	
Non-Hispanic Black	1923 (38.8%)	12671 (33.2%)	
Non-Hispanic White	1543 (31.2%)	7886 (20.6%)	
Others	478 (9.7%)	4008 (10.5%)	
Gender			0.040
Female	2050 (41.4%)	15229 (39.9%)	
Male	2902 (58.6%)	22970 (60.1%)	
BMI, kg/m2			0.082
<18.5	71 (1.4%)	564 (1.5%)	
18.5 - 24.9	1269 (25.6%)	10092 (26.4%)	
25 - 29.9	1593 (32.2%)	12698 (33.2%)	
≥30	2019 (40.8%)	14845 (38.9%)	
Diabetes status			0.065
No	2925 (59.1%)	23089 (60.4%)	
Yes	2027 (40.9%)	15110 (39.6%)	
Duration of dialysis			0.160
No	718 (14.5%)	5648 (14.8%)	
≤24 months	1049 (21.2%)	8180 (21.4%)	
25 – 60	1561 (31.5%)	12437 (32.6%)	
>60	1624 (32.8%)	11934 (31.2%)	
PRA			<0.001
0	2013 (40.7%)	21908 (57.4%)	
>0 to ≤20	876 (17.7%)	4954 (13.0%)	
>20 to ≤80	1232 (24.9%)	6867 (18.0%)	
>80	831 (16.8%)	4470 (11.7%)	
CMV status			<0.001
Low (D-/R-)	558 (11.3%)	4884 (12.8%)	
Moderate (R+)	3493 (70.5%)	25025 (65.5%)	
High (D+/R-)	901 (18.2%)	7609 (19.9%)	
Missing	0	672 (1.8%)	

Supplementary Table S2. Covariate Balance After IPTW

Covariate	Maximum Absolute Standardized Mean Difference
Donor age	0.253
Recipient age	0.127
Donor terminal creatinine	0.207
Donor BMI	0.157
Donor race/ethnicity	0.071
Donor sex	0.033
Donor hypertension	0.040
Donor diabetes	0.062
Non-heart-beating donor status	0.190
KDPI	0.050
Cause of death	0.205
CDC HIV risk status	0.075
Cold ischemia time	0.120
Recipient race/ethnicity	0.081
Recipient sex	0.052
Recipient BMI	0.057
Recipient diabetes	0.056
CMV status	0.311
Diabetes duration	0.071
PRA category	0.025
HLA mismatch	0.026

Note: Values represent the maximum absolute standardized mean difference across levels of each covariate after inverse probability of treatment weighting. An absolute standardized mean difference <0.10 is commonly considered indicative of adequate covariate balance.

Supplementary Table S3. Full IPTW-adjusted multivariable linear regression results for 1-year eGFR.

Variable	Coefficient	Lower 95% CI	Upper 95% CI	P value
Donor characteristics				
COVID-19 donor status				
Negative	Ref.			
Active	0.05	-1.09	1.19	0.93
Resolved	-0.27	-2.19	1.64	0.78
Age (per year)	-0.42	-0.44	-0.40	<0.001
Race				
Non-Hispanic White	Ref.			
Hispanic	-0.19	-0.76	0.38	0.51
Non-Hispanic Black	-0.68	-1.30	-0.07	0.03
Other	-0.70	-1.67	0.27	0.16
Sex				
Female	Ref.			
Male	3.04	2.63	3.46	<0.001
Body Mass Index				
18.5–24.9	Ref.			
<18.5	-1.16	-2.28	-0.04	0.04
25–29.9	1.15	0.66	1.64	<0.001
≥30	1.72	1.23	2.21	<0.001
Diabetes status				
No	Ref.			
Yes	-3.40	-4.13	-2.68	<0.001

Hypertension status				
No	Ref.			
Yes	-1.22	-1.71	-0.74	<0.001
Creatinine, mg/dL				
<1.0	Ref.			
1.0–1.5	-1.24	-1.73	-0.75	<0.001
>1.5	-2.34	-2.86	-1.81	<0.001
Donation after cardiac death				
No	Ref.			
Yes	3.64	3.20	4.08	<0.001
Cause of death				
Anoxia	Ref.			
Cerebrovascular	-2.95	-3.56	-2.34	<0.001
Other	-2.98	-4.10	-1.87	<0.001
Kidney Donor Profile Index				
≤0.20	Ref.			
0.21–0.35	-1.96	-2.61	-1.31	<0.001
0.36–0.85	-3.49	-4.24	-2.75	<0.001
>0.85	-3.24	-4.54	-1.94	<0.001
Risk criteria for blood-borne disease transmission				
No	Ref.			
Yes	1.32	0.85	1.79	<0.001
Recipient characteristics				

Age (per year)	-0.14	-0.16	-0.12	<0.001
Race				
Non-Hispanic White	Ref.			
Hispanic	5.73	5.14	6.33	<0.001
Black	-6.92	-7.40	-6.43	<0.001
Asian	5.07	4.33	5.81	<0.001
Sex				
Female	Ref.			
Male	1.12	0.70	1.54	<0.001
BMI, kg/m²				
18.5–24.9	Ref.			
<18.5	4.16	2.20	6.11	<0.001
25–29.9	-2.57	-3.07	-2.06	<0.001
≥30	-5.41	-5.91	-4.90	<0.001
Diabetes status				
No	Ref.			
Yes	1.08	0.66	1.50	<0.001
Duration of dialysis				
None	Ref.			
≤24 months	2.10	1.47	2.73	<0.001
25–60 months	2.43	1.83	3.03	<0.001
>60 months	2.13	1.50	2.76	<0.001
Panel reactive antibody				
0	Ref.			
>0–20	0.68	0.10	1.26	0.02

>20–80	0.32	-0.19	0.83	0.22
>80	0.05	-0.61	0.71	0.89
